# Supplementary material for: Quantification of receptor binding from response data obtained at different receptor levels: a simple individual sigmoid fitting and a unified SABRE approach
Source: Sci Rep. 2022 Nov 6;12:18833. doi: 10.1038/s41598-022-23588-w (PMC9637741; doi:10.1038/s41598-022-23588-w)
Supplement: Supplementary file 1 — Supplementary Information. [file 41598_2022_23588_MOESM1_ESM.pdf]

SUPPLEMENTARY INFORMATION

Quantification of Receptor Binding from Response Data Obtained at Different Receptor Levels: A Simple Individual Sigmoid Fitting and a Unified SABRE Approach

Peter Buchwald[*]

Department of Molecular and Cellular Pharmacology and Diabetes Research Institute, Miller School of Medicine, University of Miami, Miami, FL 33136, USA

- Tables S1–S4. Detailed parameters from all fittings
- Appendix 1. Estimation of relative efficacies assuming sigmoid response and occupancy functions

Supplementary Tables

Table S1. Parameters from fitting of data from Figure 2 with classic sigmoid model (log agonist vs response) and SABRE. Calculated parameters obtained using GraphPad Prism implementation for fitting are shown with their 95% confidence intervals (CI) and descriptors of the quality of fit (correlation coefficient, r^2, and sum of squared errors, SSE). Fit with SABRE is identical to that obtained with the standard sigmoid Hill model, e.g., "log(agonist) vs. response - Variable slope (four parameters)" model in Prism (with shared *n* and bottom restricted to 0).

Sigmoid					SABRE				
**Param.**	**Global**	**Cpd1**	**Cpd2**	**Cpd3**	**Param.**	**Global**	**Cpd1**	**Cpd2**	**Cpd3**
					ε_{R0}	= 0.00	= 0.00		
bottom	= 0.00	= 0.00			γ	= 1.00	= 1.00		
n	1.828 (1.655–2.030)	1.828 (1.655–2.030)			n	1.828 (1.655–2.030)	1.828 (1.655–2.030)		
top (E_{max})		98.90 (97.60–100.2)	76.53 (74.86–78.20)	30.81 (29.47–32.162)	ε_{Cpdi}		0.989 (0.976–)	0.765 (0.749–0.782)	0.308 (0.295–0.322)
log $EC_{50,Cpdi}$		-7.998 (-8.032–-7.964)	-6.211 (-6.255–-6.166)	-7.564 (-7.675–-7.454)	log $K_{d,Cpdi}$		-7.998 (-8.032–-7.964)	-6.211 (-6.255–-6.166)	-7.564 (-7.675–-7.454)
r^2	0.985	0.989	0.982	0.898	r^2	0.985	0.989	0.982	0.898
SSE	6589	2216	2189	2184	SSE	6589	2216	2189	2184

Table S2. Fitting results for data from Figure 3 (guinea-pig ileum preparations treated with heptyl(trimethyl)azanium, *n*-$C_7H_{15}N^+(CH_3)_3$ before and after inactivation by the irreversible antagonists dibenamine (data after ref. 18).

Parameter \ Method	Parker-Waud[18]	Individual sigmoids	SABRE
q	0.436	0.401	0.401<br>(0.204–0.598)
log K_d	-4.235	-4.108	-4.106<br>(-4.706–-3.507)

Table S3. Parameters derived from fitting with individual sigmoid functions (**A**) and SABRE (**B**) of data from Figure 4. Calculated parameters obtained from fitting using GraphPad Prism are shown with descriptors of the quality of fit (correlation coefficient, r^2, and sum of squared errors, SSE). For this fit, the Hill coefficient (slope) was fixed to unity as indicated (n = 1) in both cases (even if the sigmoid model is derived for n = 1 only). For SABRE in B, n.u. denotes parameters not used for the corresponding column.

A (sigmoids)

**Param.**	**Avg.**	**Cpd1$_{Trt_0}$**	**Cpd1$_{Trt_1}$**	**Cpd1$_{Trt_2}$**	**Cpd2$_{Trt_0}$**	**Cpd2$_{Trt_1}$**	**Cpd2$_{Trt_2}$**	**Cpd3$_{Trt_0}$**	**Cpd3$_{Trt_1}$**	**Cpd3$_{Trt_2}$**
n	= 1.00	= 1.00								
$E_{max,Cpd1}$		98.84	98.28	65.49						
log $EC_{50,Cpd1}$		-8.334	-6.822	-5.51						
log $K_{d,Cpd1}$ (eq. 14)	-4.814		*-4.589*	-5.039						
$E_{max,Cpd2}$					98.53	55.24	3.74			
log $EC_{50,Cpd2}$					-8.56	-7.298	-7.578			
log $K_{d,Cpd2}$	-7.258					-6.954	*-7.563*			
$E_{max,Cpd3}$								100.00	94.92	37.94
log $EC_{50,Cpd3}$								-7.741	-6.256	-5.091
log $K_{d,Cpd3}$	-4.930								-4.976	-4.884
q_1 (eq. 13)	0.031		0.031			0.031			0.031	
q_2 (eq. 13)	*0.0019*			0.0010			*0.0040*			0.0008
$\varepsilon_{rel\ Cpd1}$ (eq. 15)						*0.012*			*0.189*	
$\varepsilon_{rel\ Cpd1}$ (eq. 15)*						0.020			0.308	
r^2		0.986	0.988	0.968	0.985	0.963	0.113	0.986	0.985	0.919
SSE	18726	2521	2228	2316	2382	2241	2365	2758	2515	1921

B (SABRE)

<table>
<tr><th>Param.</th><th>Global</th><th>Cpd1$_{Trt_0}$</th><th>Cpd1$_{Trt_1}$</th><th>Cpd1$_{Trt_2}$</th><th>Cpd2$_{Trt_0}$</th><th>Cpd2$_{Trt_1}$</th><th>Cpd2$_{Trt_2}$</th><th>Cpd3$_{Trt_0}$</th><th>Cpd3$_{Trt_1}$</th><th>Cpd3$_{Trt_2}$</th></tr>
<tr><td>n</td><td>= 1.00</td><td colspan="9">= 1.00</td></tr>
<tr><td>γ</td><td>1982</td><td colspan="9">1982</td></tr>
<tr><td>q_1</td><td>0.0303</td><td colspan="9">0.0303</td></tr>
<tr><td>q_2</td><td>0.00096</td><td colspan="9">0.00096</td></tr>
<tr><td>ε_{Cpd1}</td><td>1.000</td><td colspan="3">1.000</td><td colspan="6"></td></tr>
<tr><td>log $K_{d,Cpd1}$</td><td>-5.031</td><td colspan="3">-5.031</td><td colspan="6"></td></tr>
<tr><td>ε_{Cpd2}</td><td>0.021</td><td colspan="3"></td><td colspan="3">0.021</td><td colspan="3"></td></tr>
<tr><td>log $K_{d,Cpd2}$</td><td>-6.949</td><td colspan="3"></td><td colspan="3">-6.949</td><td colspan="3"></td></tr>
<tr><td>ε_{Cpd3}</td><td>0.313</td><td colspan="6"></td><td colspan="3">0.313</td></tr>
<tr><td>log $K_{d,Cpd3}$</td><td>-4.955</td><td colspan="6"></td><td colspan="3">-4.955</td></tr>
<tr><td>r^2</td><td>0.986</td><td>0.985</td><td>0.988</td><td>0.968</td><td>0.985</td><td>0.963</td><td>0.106</td><td>0.986</td><td>0.985</td><td>0.918</td></tr>
<tr><td>SSE</td><td>21437</td><td>2596</td><td>2229</td><td>2319</td><td>2424</td><td>2242</td><td>2384</td><td>2787</td><td>2516</td><td>1941</td></tr>
</table>

Table S4. Parameters derived from fitting with individual sigmoid functions (**A**) and SABRE (**B**) of data from Figure 5 (muscarinic activity in rabbit myocardium). As before, calculated parameters obtained from fitting using GraphPad Prism are shown with descriptors of the quality of fit (r^2 and SSE). For the sigmoid fitting, to obtain a better fit, which is also more consistent with the unified SABRE fit, the Hill slope n was released from unity and constrained to a single shared value across all groups.

A (sigmoids)

Param.	Avg.	OxtrM$_{BCM0}$	OxtrM$_{BCM1}$	OxtrM$_{BCM10}$	Oxtr$_{BCM0}$	Oxtr$_{BCM1}$	Oxtr$_{BCM10}$	BM5$_{BCM0}$	BM5$_{BCM1}$	BM5$_{BCM10}$
n	0.772	0.772								
$E_{max,OxtM}$		98.90	85.04	31.41						
log $EC_{50,OxtrM}$		-6.404	-5.936	-5.650						
log $K_{d,cOxtrM}$ (eq. 14)	-5.349		-5.233	-5.509						
$E_{max,Oxtr}$					95.66	77.28	24.08			
log $EC_{50,Oxtr}$					-6.814	-6.420	-6.379			
log $K_{d,Oxtr}$	-6.036					-5.875	-6.295			
$E_{max,BM5}$								52.78	26.63	7.49
log $EC_{50,BM5}$								-6.597	-6.597	-6.156
log $K_{d,BM5}$	-6.290								-6.596	-6.112
q_1 (eq. 13)	0.374		0.293			0.326			0.504	
q_2 (eq. 13)	0.067			0.056			0.092			0.051
$\varepsilon_{rel\ OxtrM}$ (eq. 15)						0.642			0.116	
r^2	0.994	0.997	0.994	0.971	0.989	0.986	0.969	0.977	0.930	0.855
SSE	474.1	29.2	56.0	32.0	88.1	97.3	21.2	72.8	65.5	12.1

B (SABRE)

Param.	Global	OxtrM$_{BCM0}$	OxtrM$_{BCM1}$	OxtrM$_{BCM10}$	Oxtr$_{BCM0}$	Oxtr$_{BCM1}$	Oxtr$_{BCM10}$	BM5$_{BCM0}$	BM5$_{BCM1}$	BM5$_{BCM10}$
n	0.777	0.777								
γ	7.276	7.276								
q_1	0.406	0.406								
q_2	0.065	0.065								
ε_{OxtrM}	1.000	1.000								
log $K_{d,OxtrM}$	-5.273	-5.273								
ε_{Oxtr}	0.747				0.747					
log $K_{d,Oxtr}$	-5.853				-5.853					
ε_{BM5}	0.128							0.128		
log $K_{d,BM5}$	-6.314							-6.314		
r^2	0.993	0.997	0.993	0.964	0.989	0.986	0.927	0.977	0.919	0.749
SSE	550.0	33.7	60.2	40.4	91.6	100.2	50.9	76.0	76.2	20.9

Appendix 1. Estimation of Relative Efficacies Assuming Sigmoid Response and Occupancy Functions

Assuming straightforward hyperbolic functions (i.e., n = 1) for two compounds of different efficacy (E_{max}) and potency (EC_{50}), the effect E (response) depends on ligand concentration [L] as:

$$E_1 = E_{max,1} \frac{[L_1]}{[L_1]+EC_{50,1}} \tag{A1}$$

$$E_2 = E_{max,2} \frac{[L_2]}{[L_2]+EC_{50,2}} \tag{A2}$$

With this assumption, the ratio of ligand concentrations [L_1] and [L_2] causing the same effect E, which is small to ensure that is obtainable even with the weak partial agonists (see Figure below), can be obtained following the approach used to obtain the ratio of *equiactive molar ratios* by Ehlert and co-workers (EAMR; i.e., ratio of molar concentrations that produce the same response at low enough concentrations – later termed *intrinsic relative activity*, RA_i)[23,24]:

$$E_{max,1} \frac{[L_1]}{[L_1]+EC_{50,1}} = E_{max,2} \frac{[L_2]}{[L_2]+EC_{50,2}} \tag{A3}$$

Rearranging to get the ratio of ligand concentrations:

$$\frac{[L_2]}{[L_1]} = \frac{E_{max,1}EC_{50,2}}{[L_1](E_{max,2}-E_{max,1})+E_{max,2}EC_{50,1}} \tag{A4}$$

which at sufficiently low ligand concentrations approaches the EAMR[23]:

$$\frac{[L_2]}{[L_1]} \xrightarrow{[L]\to 0} \frac{E_{max,1}EC_{50,2}}{E_{max,2}EC_{50,1}} = \frac{E_{max,1}/EC_{50,1}}{E_{max,2}/EC_{50,2}} \tag{A5}$$

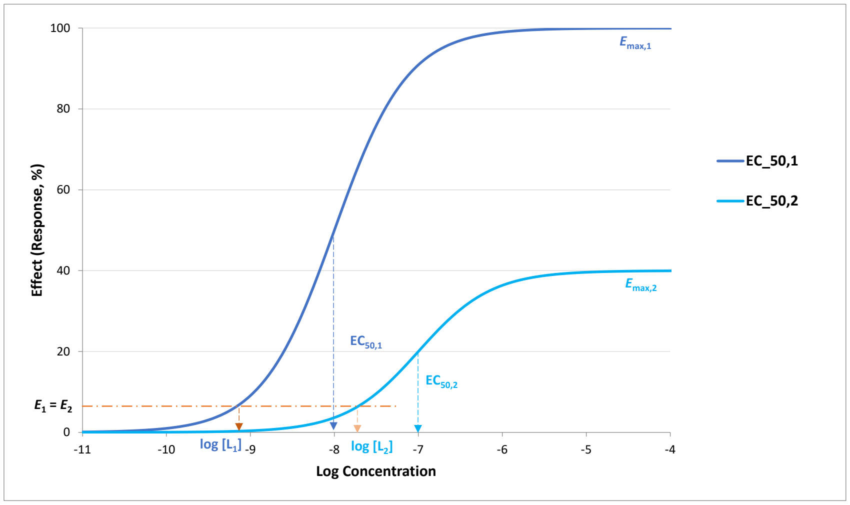

This E_{max}/EC_{50} ratio is a frequently used basis of bias quantification, i.e., a logarithmic bias factor is obtained by calculating the shift $\Delta\Delta\log(E_{max}/EC_{50})$, i.e., $\Delta_{\text{Test vs Ref}}\Delta_{\text{Pathway 1 vs 2}}\log(E_{max}/EC_{50})$: $(\log(E_{max,P_1,L}/EC_{50,P_1,L})-\log(E_{max,P_2,L}/EC_{50,P_2,L})) - ((\log(E_{max,P_1,Lref}/EC_{50,P_1,Lref})-\log(E_{max,P_2,Lref}/EC_{50,P_2,Lref}))$[25-28]. Comparing the change on E_{max}/EC_{50} is replaced with that of the "transduction coefficient" τ/K_D if the operational model is used or it can also be replaced with $\varepsilon\gamma/K_d$ if SABRE is used (assuming n = 1; see Appendix 2 of ref. 1 and eq. 23 here).

To compare the relative efficacies of two compounds in the same assay fitted with sigmoid responses, one can follow the simple original approach by Furchgott, and assume that at conditions that produce equal responses, the ratio of efficacies (i.e., relative efficacies) is the inverse of the ratio of occupied receptors producing it[5]. Thus, with the notation used by Furchgott:

$$\frac{\epsilon_2}{\epsilon_1} = \frac{[RA_1]/[R_t]}{[RA_2]/[R_t]} \qquad (A6)$$

Or with the notation used for SABRE:

$$\frac{\varepsilon_2}{\varepsilon_1} = \frac{[R_{occup,1}]/[R_{tot}]}{[R_{occup,2}]/[R_{tot}]} = \frac{f_{occup,1}}{f_{occup,2}} \qquad (A7)$$

If one assumes that receptor occupancy follows a classic law of mass action and, hence, a sigmoid response function (on log scale) characterized by the K_d dissociation constant (which is determined here from responses measured at different receptor levels and fitted with individual functions, see eq. 14), f_{occup} for compound i is described by:

$$f_{occup,i} = \frac{[L_i]}{[L_i]+K_{d,i}} \qquad (A8)$$

Thus, the ratio of occupancies as a function of ligand concentrations is

$$\frac{f_{occup,1}}{f_{occup,2}} = \frac{[L_1]}{[L_2]}\frac{[L_2]+K_{d,2}}{[L_1]+K_{d,1}} \qquad (A9)$$

At low enough concentrations, which are assumed here in order to have low enough effects (see Figure and eq. A5), $[L_1] << K_{d,1}$ and $[L_2] << K_{d,2}$; thus, this ratio becomes:

$$\frac{f_{occup,1}}{f_{occup,2}} \xRightarrow{[L]\to 0} \frac{[L_1]}{[L_2]}\frac{K_{d,2}}{K_{d,1}} \qquad (A10)$$

Using this in the equation for relative efficacies derived above (A7) and then introducing the ligand concentrations that produce the same (small) effect from eq. A5:

$$\frac{\varepsilon_2}{\varepsilon_1} = \frac{f_{occup,1}}{f_{occup,2}} = \frac{[L_1]}{[L_2]}\frac{K_{d,2}}{K_{d,1}} = \frac{E_{max,2}/EC_{50,2}}{E_{max,1}/EC_{50,1}}\frac{K_{d,2}}{K_{d,1}} \quad (A11)$$

Which can be rearranged as

$$\frac{\varepsilon_2}{\varepsilon_1} = \frac{E_{max,2}\frac{K_{d,2}}{EC_{50,2}}}{E_{max,1}\frac{K_{d,1}}{EC_{50,1}}} \quad (A12)$$

Therefore, comparing relative efficacies can be done by comparing $E_{\mathrm{max}}{\cdot}K_{\mathrm{d}}/\mathrm{EC}_{50}$ values. The same relationship (eq. A12, eq. 15) can be obtained much more easily from the SABRE approach by using eq. 26, i.e.,

$$\varepsilon\gamma = E_{max}\frac{K_d}{EC_{50}} \quad (A13)$$

to express the ratio of efficacies (ε) and noticing that γ is eliminated as the pathway and, hence, the pathway amplifications are the same.
